# Oropouche virus infection: an emerging arboviral threat in Asia

**DOI:** 10.7717/peerj.21712

**Published:** 2026-09-15

**Authors:** Norhafizah Saari, Rafidah Hanim Shueb

**Affiliations:** Department of Medical Microbiology and Parasitology, School of Medical Sciences, Health Campus, Universiti Sains Malaysia, Kubang Kerian, Kelantan, Malaysia

**Keywords:** Oropouche virus, Arbovirus, Asian country, Spread

## Abstract

**Background:**

Oropouche virus (OROV), a neglected arbovirus of the *Orthobunyavirus* genus within the *Peribunyaviridae* family, has historically been confined to South and Central America. Recent epidemiological changes, including geographic expansion, increasing incidence, exported travel-associated cases, and reports of neurological and congenital complications, have raised concern regarding its potential for wider dissemination. Although no confirmed cases have been reported in Asia to date, future emergence remains biologically and ecologically plausible due to interconnected virological, ecological, and anthropogenic factors.

**Methodology:**

This narrative literature review was conducted using PubMed, Scopus, and Web of Science from September 2025 to May 2026. A total of 7,939 records were initially identified, and 105 relevant sources were selected based on their relevance to OROV virology, transmission dynamics, clinical manifestations, evolutionary potential, environmental drivers, and possible emergence in Asia.

**Results:**

Current evidence shows that OROV has progressed from a regionally neglected arbovirus to an emerging preparedness concern. Its segmented RNA genome enables reassortment, supporting viral adaptability and the emergence of strains such as OROVBR-2015–2024, which have been associated with enhanced transmission potential and more severe clinical outcomes. In Asia, potential emergence would likely result from multiple converging factors rather than a single determinant. These include the presence of diverse biting midges and mosquitoes that require vector competence assessment, climate conditions favourable to vector persistence, land-use change, urbanisation, increased wildlife–livestock–human contact, and frequent international travel. Clinical overlap with dengue, Zika, and chikungunya may further delay recognition, particularly in settings where OROV molecular testing is not routinely available.

**Conclusion:**

OROV emergence in Asia is not inevitable but remains a credible preparedness concern. Proactive surveillance, diagnostic readiness, vector competence studies, and regional preparedness are needed to enable early detection and reduce the risk of introduction and establishment.

## Introduction

Arthropod-borne viruses (arboviruses) are emerging and re-emerging pathogens transmitted by hematophagous vectors, including mosquitoes, ticks, flies, and biting midges, and continue to impose substantial public health burdens, particularly in tropical and subtropical regions (Ribas Freitas et al., 2025; Siew et al., 2025b; Yoosuf et al., 2025). Although dengue, Zika, and chikungunya viruses have received greater global attention because of their epidemic potential and wide clinical spectrum, less-recognised arboviruses such as OROV are increasingly gaining attention as emerging threats.

OROV, the causative agent of Oropouche fever, was first identified in Trinidad and Tobago in 1955 and has caused recurrent epidemics, especially in Brazil and Peru, with more than half a million infections reported (Yoosuf et al., 2025). Its transmission is maintained through interconnected urban and sylvatic cycles, primarily involving *Culicoides* biting midges, mosquitoes, and wild vertebrate hosts (Bandeira et al., 2024; Gallichotte, Ebel & Carlson, 2025; McGregor, Connelly & Kenney, 2021; Pinheiro et al., 1981; Yoosuf et al., 2025). Since late 2022, OROV epidemiology has shifted markedly, with transmission expanding beyond the Amazon Basin into previously unaffected areas of South America and the Caribbean, including Cuba (Ribas Freitas et al., 2025). In Brazil, the 2024 outbreak showed a 58.8-fold increase in incidence compared with the annual median from 2015 to 2023 and demonstrated widespread national distribution rather than the historically localised pattern in northern regions (Scachetti et al., 2025).

This resurgence has been accompanied by increasing reports of severe disease, shifting OROV from a largely self-limiting febrile illness to neurological complications, including meningitis, encephalitis, and Guillain-Barré syndrome, as well as adult fatalities and adverse perinatal outcomes linked to vertical transmission (De Armas Fernández et al., 2024; González-Quevedo, Lestayo O’Farrill & Becquer, 2025; WHO, 2024b; WHO, 2025a). In 2024, 16,239 confirmed cases and four deaths were reported across the Americas, with further adult fatalities and neurological complications documented into 2025, underscoring the growing clinical and public health significance of OROV (CDC, 2025b; De Armas Fernández et al., 2024).

Molecular studies suggest that this changing epidemiological and clinical profile may be associated with novel reassortant lineages, including OROVBR-2015–2024, which may exhibit altered pathogenicity and transmissibility (Naveca et al., 2024; Schwartz, 2025). These developments highlight the evolutionary plasticity of OROV and its capacity to extend beyond traditional endemic boundaries. Therefore, OROV should no longer be viewed solely as a localised Amazonian pathogen but as an emerging arboviral threat of broader global relevance.

A recent review by Bai et al. (2025) have focused on retrospective outbreak patterns and molecular characteristics of OROV in the Americas. However, vector ecology, zoonotic reservoirs, environmental change, and anthropogenic drivers beyond OROV’s established range, especially in Asia, remain insufficiently integrated into prospective risk assessment.

This review provides an integrated synthesis of the potential emergence and risk factors of OROV, with emphasis on viral adaptability, vector ecology, zoonotic risks, and environmental drivers relevant to its plausible emergence in Asia. It aims to support researchers, clinicians, entomologists, epidemiologists, and public health policymakers by identifying the biological and ecological conditions that could facilitate OROV introduction, detection, and potential establishment in the region.

## Survey Methodology

A structured literature search was conducted using PubMed, Scopus and Web of Science to identify studies relevant to OROV infection and its potential emergence in Asia. The search was conducted from September 2025 to May 2026. Search terms included “Oropouche virus”, “OROV”, “arbovirus”, “epidemiology”, “zoonotic transmission”, “clinical manifestations”, “climate change”, “Asia”, and “emerging infectious disease”. The search terms were combined using the Boolean operators AND and OR. The initial search identified 7,939 records, which were screened for duplicates before title and abstract screening. Full-text assessment was then performed to determine eligibility. A total of 105 sources were ultimately included based on their relevance to OROV virology, transmission dynamics, clinical manifestations, evolutionary potential, environmental drivers, and preparedness for possible emergence in Asia. Eligible sources included peer-reviewed articles, epidemiological studies, surveillance reports, and authoritative public health documents from the World Health Organization (WHO), Pan American Health Organization (PAHO), and Centers for Disease Control and Prevention (CDC). Publications were excluded if they were not directly relevant to OROV or arboviral emergence, lacked sufficient methodological or epidemiological detail, were conference abstracts without full data, or were non-English articles without accessible translation. Reference lists of key articles were also manually screened. The included publications were organised thematically across virology, transmission dynamics, vector ecology, zoonotic interfaces, clinical manifestations, environmental drivers, and public health preparedness. This integrative approach was used to identify knowledge gaps and evaluate conditions that may influence the potential emergence of OROV in Asia.

## Global and Temporal Prevalence

### Historical distribution (pre-2023)

OROV has historically been confined to the Amazon Basin, with Brazil serving as the primary hotspot of activity where both sporadic cases and periodic outbreaks have been documented (González-Quevedo, Lestayo O’Farrill & Becquer, 2025). Since its discovery in Trinidad and Tobago in 1955, OROV has been implicated in more than half a million reported infections across South and Central America by 2005 (PAHO, 2024a; PAHO, 2024b). Beyond Brazil, outbreaks were reported in Panama (1989), Argentina’s Jujuy Province (2005), Haiti (2014), and French Guiana (2020) (CDC, 2024; PAHO, 2025b; WHO, 2016). Despite its recurring activity, OROV was often considered a neglected arbovirus, overshadowed by higher-profile pathogens such as dengue and Zika (Nascimento et al. 2020). Data and serological studies have revealed that OROV circulation extended into Colombia, Peru, and Ecuador, particularly in forested and peri-urban regions of the Amazon Basin, where ecological conditions favoured midge-borne transmission (Okesanya et al., 2025). In these areas, the virus was frequently underreported due to limited diagnostic capacity and overlapping clinical presentations with other febrile illnesses. Additionally, evidence of OROV antibodies in non-human primates and sloths suggested a broader enzootic reservoir that may have facilitated silent transmission cycles in regions not previously recognized as endemic (Toews et al., 2025). This cryptic spread, coupled with the virus’s adaptability to urban environments, laid the groundwork for its later emergence in new territories (Okesanya et al., 2025).

### Recent epidemiological trends (2023–2025)

**Figure 1:**
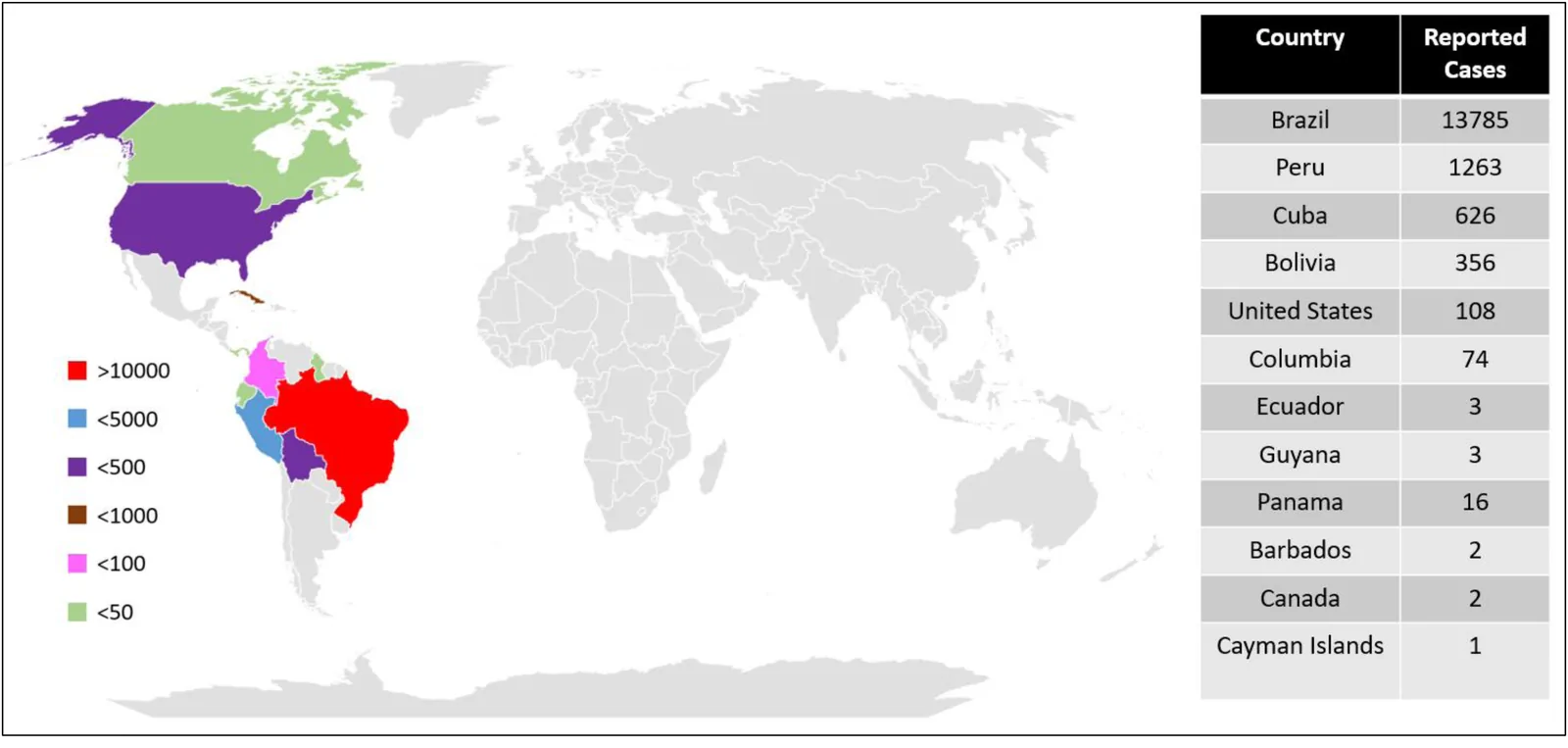
Geographic distribution and epidemiological patterns of OROV infection in the Americas. The geographic distribution of reported OROV cases in the Americas. Colour-shaded countries representing cumulative case categories ranging from <50, to >10,000 cases. The accompanying table summarises reported case numbers by country. The distribution shows the highest burden in South America, particularly Brazil and neighbouring endemic regions, while also indicating geographic expansion into the Caribbean and travel-associated detection in North America. This pattern underscores the need for strengthened surveillance, early diagnostic capacity, and preparedness in both endemic and non-endemic regions.Created in: Microsoft Excel powered by Bing Maps.

A marked epidemiological shift has occurred since late 2022, becoming clearly evident during 2023–2025 through rapid expansion in both scale and geographic distribution (Table 1 & Fig. 1). Therefore, 2023 was used as the cut-off point to distinguish the historically localised and underreported phase of OROV transmission from the recent expansion phase. Between 2023 and 2024, Brazil experienced widespread transmission across all 24 states, with outbreaks extending beyond the Amazon Basin into previously unaffected regions such as Bahia, Espírito Santo, and Rio de Janeiro, where fatal cases were reported for the first time (Benitez et al., 2024; Sah et al., 2024). The Amazon Basin and its tributaries remain a major ecological hotspot for arboviruses due to favourable climatic conditions, including high humidity, temperature, and vector abundance.

**Table 1:** Geographical distribution, case burden, and transmission dynamics of OROV infection in the Americas (1955–2025).

**Country**	**Reported cases**	**Prevalence**	**Genotype/ Lineage**	**Vector**	**Transmission type**	**Epidemiological**	**References**
Brazil	13,785 (2023–2024)	Major endemic; outbreak across 24 states; incidence increased ∼58.8-fold *vs* 2015–2023 median	OROVBR-2015– 2024 (reassortant)	*Culicoides paraensis* (primary); *Culex quinquefasciatus* (suspected)	Endemic & epidemic	First nationwide spread; fatal cases reported; shift from localized Amazonian transmission	PAHO, 2024a; PAHO, 2024b; PAHO, 2025a; PAHO, 2025b; WHO, 2024a; WHO, 2024b
Peru	1,263	Endemic in Amazon Basin; recurrent outbreaks in rural–peri-urban interface	Genotype II	*Culicoides paraensis* (primary)	Endemic	Sustained sylvatic-to-urban transmission cycles	PAHO, 2025b; WHO, 2016
Bolivia	356	Localized outbreaks in tropical lowlands	Not specified	*Culicoides spp.*; mosquitoes (suspected)	Local transmission	Linked to ecological suitability and vector adaptation	CDC, 2025b; PAHO, 2025b
Cuba	626 (2024)	1st major Caribbean outbreak; widespread municipal transmission	Brazilian lineage (OROVBR- 2015–2024)	*Culicoides paraensis* (primary)	Local transmission	First Caribbean emergence; neurological complications reported	De Armas Fernández et al., 2024; PAHO, 2024b; PAHO, 2025b
Colombia	74	Sporadic outbreaks in Amazonian regions	Not specified	*Culicoides spp.*	Local transmission	Underreported due to diagnostic limitations	PAHO, 2025b; PAHO, 2025a
Ecuador	3	Limited cases; sporadic transmission	Not specified	Not specified	Local transmission	Low reported burden	PAHO, 2024a; PAHO, 2024b
Guyana	3	Small cluster cases	Not specified	Not specified	Local transmission	Emerging activity	PAHO, 2024a; PAHO, 2024b
Panama	16	Historical and recent transmission	Genotype III	*Culicoides spp.*	Endemic/local	Longstanding circulation	PAHO, 2024a; PAHO, 2024b
Barbados	2 (2024)	Newly reported Caribbean cases	Not specified	Likely *Culicoides spp.*	Local transmission	Expansion beyond mainland Americas	PAHO, 2024a; PAHO, 2024b
Venezuela	Emerging (2025)	Re-emergence after decades	Not specified	Not specified	Local transmission	1st confirmed cases after long absence	Cruz et al., 2025
United States	108	Reported Imported cases rate	Not applicable	Not applicable	Imported	Travelers returning from Cuba and endemic regions	CDC, 2024; CDC, 2025c; CDC, 2025b
Canada	2	Reported Imported cases rate	Not applicable	Not applicable	Imported	Travel-associated infections	CDC, 2024; CDC, 2025c; CDC, 2025b
Europe (Spain, Italy, Germany)	Multiple cases	Reported Imported cases rate	Not applicable	Not applicable	Imported	Linked to travel from endemic regions	Salvato, 2025

Genomic surveillance has identified the emergence of a reassortant lineage, OROVBR-2015–2024, which exhibits enhanced replication efficiency and reduced neutralisation by antibodies from earlier strains, providing a virological basis for its rapid spread and increased epidemic potential (Benitez et al., 2024). Concurrently, OROV has expanded into new geographic regions across South America and the Caribbean. Cuba reported its first confirmed case in May 2024, linked to a Brazilian lineage introduced from Acre State, followed by widespread transmission across 99 municipalities (Benitez et al., 2024; De Armas Fernández et al., 2024). Barbados and Guyana documented their initial human cases later that year, while Venezuela reported its first autochthonous cases in March 2025, representing a significant re-emergence after decades of epidemiological silence (Cruz et al., 2025). These patterns are consistent with broader regional expansion trends observed during the 2023–2025 outbreak, with increasing case numbers and geographic spread beyond traditional endemic areas.

The globalisation of OROV has also been facilitated by international travel, resulting in imported cases in Europe and North America. Notably, 21 cases were identified among United States travellers returning from Cuba in 2024, with additional cases reported in Spain, Italy, Germany, Canada, and the United States, all associated with travel to endemic regions (Salvato, 2025). In Asia, Thailand was classified as “screened/ 0 case” based on 1,374 OROV-negative serum samples from acute febrile illness patients (Khongwichit et al., 2025). These findings highlight the need to distinguish between countries with active screening but no detected cases and those with no surveillance data. Collectively, this unprecedented expansion beyond the traditional Amazonian range highlights the urgent need for strengthened surveillance, genomic monitoring, and preparedness strategies in both endemic and non-endemic regions.

## OROV Transmission

OROV transmission occurs through two interconnected cycles, the urban and sylvatic (jungle) cycles with human movement facilitating interactions between these transmission systems. Rather than operating as isolated pathways, these cycles form a fluid ecological continuum where vector biology, wildlife reservoirs, human behaviour, and environmental drivers interact to shape regional epidemiological patterns.

In the urban cycle, the primary vector is *Culicoides paraensis*, a biting midge that is widely distributed in endemic regions such as Brazil. Transmission is mediated by adult female midges, which require blood meals for egg production, enabling viral dissemination throughout their 20 to 30-day lifespan (Bandeira et al., 2024; Pinheiro et al., 1981; Yoosuf et al., 2025). Other *Culicoides* species, including *Culicoides sonorensis*, have been investigated as potential vectors; they demonstrated high experimental infection and dissemination rates, although their transmission efficiency appears comparatively lower (McGregor, Connelly & Kenney, 2021).

In addition to biting midges, several mosquito species have been implicated as secondary vectors, including *Culex quinquefasciatus*, *Aedes aegypti*, and *Aedes serratus*. Among these, *Culex quinquefasciatus* exhibits limited competence for OROV transmission, suggesting a relatively minor role compared with *Culicoides* midges (McGregor, Connelly & Kenney, 2021; Pinheiro et al., 1981).

The sylvatic cycle involves the circulation of OROV among wild vertebrate hosts within the forest ecosystems. Documented hosts include non-human primates such as capuchin and howler monkeys, sloths, and potentially avian species, with transmission mediated by mosquitoes including *Coquillettidia venezuelensis*, *Aedes serratus*, and *Culex quinquefasciatus* (Gallichotte, Ebel & Carlson, 2025). Serological surveys have detected haemagglutination inhibiting antibodies against OROV in multiple bird families, including Formicariidae, Troglodytidae, Cuculidae, Fringillidae, Dendrocolaptidae, Tyrannidae, Vireonidae, Thraupidae, and Pipridae, indicating exposure in avian populations (Yoosuf et al., 2025). However, the role of birds in OROV transmission remains incompletely understood. Current evidence does not support their classification as primary reservoirs; instead, they are more likely to play a secondary or ecological role in maintaining viral circulation and facilitating interactions between vectors and other vertebrate hosts.

Direct human-to-human transmission of OROV has not been previously documented. Nevertheless, recent findings have expanded the understanding of viral shedding dynamics, with OROV RNA detected in the saliva and urine of infected patients (Nascimento et al., 2020). In addition, vertical transmission has been confirmed, and detection of viral RNA in semen suggests the possibility of sexual transmission, although this remains to be conclusively established (Iglói et al., 2025; Schwartz, 2025). It is worth noting that the apparent rise in OROV reports may also partly reflect improved diagnostic capacity, pathogen surveillance, and public health awareness after the COVID-19 pandemic. Thus, the recent increase should be interpreted as the product of both true ecological and epidemiological expansion and enhanced detection, rather than surveillance artefact alone.

### Clinical manifestation and management

Oropouche fever typically presents as an acute febrile illness with abrupt onset 3–10 days after the bite of an infected arthropod, although the incubation period may extend up to 12 days (Morrison et al., 2024; Wesselmann et al., 2024; WHO, 2016). The illness is commonly characterised by high fever, severe headache, prostration, chills, myalgia, and arthralgia (Morrison et al., 2024; Wang et al., 2025). Headache is often intense and parieto-occipital in distribution, while arthralgia generally lacks the inflammatory features typically observed in chikungunya infection (Tortosa et al., 2024).

Other systemic manifestations may include conjunctival injection, retro-orbital pain, photophobia, dizziness, low back pain, abdominal discomfort, nausea, vomiting, diarrhoea, and maculopapular rash (Anderson et al., 1961; Morrison et al., 2024; Mourãão et al., 2009; Wang et al., 2025). Haemorrhagic symptoms, including petechiae, epistaxis, gingival bleeding, melena, and menorrhagia, have also been reported in approximately 16% of patients (Mourãão et al., 2009). The acute phase usually lasts two to five days, and most patients recover within several days to one month (Da Rosa et al., 2017; (Wesselmann et al., 2024). A biphasic course may occur in 60–70% of patients, with symptom recurrence 7–10 days after initial onset, likely reflecting recrudescence rather than reinfection (Castilletti et al., 2024). Laboratory findings may include leukopenia, lymphopenia, mild transaminase elevation, and occasional thrombocytopenia (Pastula, Beckham & Tyler, 2024).

### Severe complications

Although Oropouche fever was historically considered a high-morbidity but low-mortality illness, recent outbreaks have shown an expanding spectrum of severe disease. Neurological involvement is the most prominent complication, including aseptic meningitis, encephalitis, meningoencephalitis, encephalopathy, vertigo, nystagmus, diplopia, dysgeusia, hearing loss, and seizures. Aseptic meningitis usually appears during the second week of illness and may prolong recovery (Bastos et al., 2012; Pastula, Beckham & Tyler, 2024; Vernal, Martini & da Fonseca, 2019; Yoosuf et al., 2025). In a Cuban case series, encephalitis was reported in 29% of 38 patients, while cerebrospinal fluid findings in meningoencephalitis commonly showed lymphocytic pleocytosis, normal glucose, and elevated protein levels (Bello-Rodríguez et al., 2025; Pastula, Beckham & Tyler, 2024).

Guillain–Barré syndrome has also been associated with OROV infection. During the 2024 Cuban outbreak, OROV RNA was detected in serum or cerebrospinal fluid from 65 patients with Guillain–Barré syndrome, 62% of whom reported a preceding undifferentiated febrile illness one to two weeks earlier (De Armas Fernández et al., 2024; De Armas Fernández et al., 2025). Severe illness may also involve haemorrhagic symptoms, leukopenia, lymphopenia, elevated C-reactive protein, mild liver enzyme elevation, and rare thrombocytopenia (Da Rosa et al., 2017; Mourãão et al., 2009; Pastula, Beckham & Tyler, 2024; Wang et al., 2025). Reports of deaths among previously healthy young adults in Brazil further suggest possible changes in virulence and support continued surveillance of OROV pathogenicity (Bandeira et al., 2024; CDC, 2024; Morrison et al., 2024).

### Impact on pregnant women and fetal outcomes

Vertical transmission has become a major concern in recent OROV outbreaks. Although maternal illness appears similar to infection in non-pregnant individuals, infection during pregnancy may pose distinct fetal risks (Schwartz, 2025). OROV RNA has been detected in mothers, infants, and fetal tissues, with maternal infection linked to miscarriage, fetal demise, and congenital abnormalities (Cola et al., 2025; Schwartz, Dashraath & Baud, 2024).

A possible association between OROV infection and miscarriage was first reported during Brazilian outbreaks in the 1980s (Borborema et al., 1982). Stronger evidence emerged in 2024 in Pernambuco State, where maternal infection at 30 weeks of gestation was followed by fetal demise two weeks later, with OROV RNA detected in fetal tissue (Martins-Filho, Carvalho & dos Santos, 2024; Schwartz, Dashraath & Baud, 2024; WHO, 2024b). Subsequent reports described miscarriage, intrauterine fetal death, microcephaly, ventriculomegaly, agenesis of the corpus callosum, craniosynostosis, porencephalic changes, skeletal abnormalities, growth restriction, hydrops fetalis, oligohydramnios, cerebral atrophy, intracranial cysts, spinal cord thinning, posterior fossa malformations, and ocular abnormalities (Das Neves Martins et al., 2025; Nielsen-Saines & Brasil, 2025; Ribeiro et al., 2025; Schwartz, 2025; Schwartz, Dashraath & Baud, 2024; Srivastava et al., 2024).

In a series of 73 pregnancies with OROV infection, outcome data were available for 15 cases. Two infections occurred during the first trimester: one ended in miscarriage, whereas the other resulted in a live birth with corpus callosum anomalies. By contrast, all 13 third-trimester infections were associated with non-anomalous live births (Cola et al., 2025). Although evidence remains limited, these findings suggest that earlier gestational infection may carry greater risk of pregnancy loss or central nervous system anomalies. The overlap with features of congenital Zika syndrome highlights the need for pregnancy-focused surveillance, timely diagnosis, and preventive guidance in affected and at-risk regions (Cola et al., 2025; Das Neves Martins et al., 2025; Ribeiro et al., 2025; Schwartz, Dashraath & Baud, 2024).

### Diagnostic approaches for OROV infection

Diagnosis of OROV infection is challenging because the infection clinically overlaps with dengue, Zika, chikungunya, and other arboviral diseases. Laboratory confirmation is therefore essential, especially in areas where multiple arboviruses co-circulate and clinical assessment alone is unreliable (Table 2). During the acute viraemic phase, reverse transcription polymerase chain reaction (RT-PCR) or reverse transcription quantitative polymerase chain reaction (RT-qPCR) is the preferred diagnostic method because viral RNA is most detectable during the first few days after symptom onset (CDC, 2025b; CDC, 2025a; PAHO, 2024b; WHO, 2025b).

**Table 2:** Comparative overview of arboviral infections with overlapping clinical features relevant to OROV.

**Pathogen/ Disease**	**Clinical manifestations**	**Diagnostic methods**	**Key distinguishing features**	**Serological cross-reactivity with OROV**	**Reference**
Oropouche virus (OROV)	Acute febrile illness, high fever, headache, myalgia, arthralgia, rash; biphasic fever; possible neurological complications (meningitis, encephalitis, Guillain– Barré syndrome)	RT-PCR, serology	Biphasic course with relapse; neurological involvement increasingly reported; typically, non-fatal but severe cases documented	Limited/ not well established	De Armas Fernández et al., 2024; Morrison et al., 2024; Yoosuf et al., 2025
Dengue virus	High fever, severe headache, retro-orbital pain, myalgia, rash, thrombocytopenia, haemorrhagic manifestations	RT-PCR, serology	Haemorrhagic complications; plasma leakage; thrombocytopenia prominent	No cross- reactivity (different virus family)	CDC, 2025d; WHO, 2025b
Chikungunya virus	High fever, severe polyarthralgia, headache, rash	RT-PCR, serology	Persistent joint pain lasting weeks to months	No cross- reactivity	WHO, 2025b
Yellow fever virus	Fever, myalgia, jaundice, haemorrhage in severe cases	RT-PCR, serology	Hepatic involvement leading to jaundice (“yellowing”)	No cross- reactivity	CDC, 2025a
Zika virus	Low-grade fever, rash, arthralgia, conjunctivitis; neurological complications; congenital abnormalities	RT-PCR, serology	Mild symptoms; strong association with congenital microcephaly	No cross- reactivity	Musso, Ko & Baud, 2019; WHO, 2025b
Mayaro virus	Fever, rash, myalgia, arthralgia	RT-PCR, serology	Similar to chikungunya; prolonged joint symptoms possible	No cross- reactivity	Silva-Ramos et al., 2023

Serological assays, including enzyme-linked immunosorbent assay (ELISA) based detection of OROV-specific IgM and IgG, are useful later in infection after viraemia declines, but cross-reactivity with related *Orthobunyaviruses* may limit specificity and require confirmatory testing (Bai et al., 2025; WHO, 2016). Virus isolation is valuable for research and reference laboratories but is unsuitable for routine frontline diagnosis because it requires specialised facilities, technical expertise, and several days to generate results (PAHO, 2024a). In OROV propagation and experimental characterisation, Vero cells are commonly used because their interferon-deficient background enhances viral permissiveness (De Almeida et al., 2025; Osada et al., 2014). Human serum-derived OROV has been demonstrated to produce cytopathic effects in Vero cell monolayers within 48–72 h, with replication confirmed by plaque assay, RT-qPCR, and transmission electron microscopy (De Almeida et al., 2025).

Animal models are also important for understanding pathogenesis and supporting mechanistic studies, although they are not diagnostic tools. Experimental studies have shown that neonatal BALB/c mice exhibited fatal neurological disease with viral replication in brain neurons, while C57BL/6J pregnancy models demonstrated maternal tissue replication and placental infection, providing insights into neurotropism and vertical transmission (Gunter et al., 2026; Santos et al., 2012). Hence, given the risk of underdiagnosis in non-endemic regions, OROV should be integrated into arboviral diagnostic panels, particularly in Asia, where dengue-like illness may obscure imported or emerging infections (WHO, 2024a; WHO, 2025a).

### Clinical management and supportive care

Currently, no specific antiviral therapy is approved for OROV infection; therefore, treatment remains supportive. In most cases, infections are self-limiting and can be managed with antipyretics, analgesics, hydration, and clinical monitoring (CDC, 2025b; WHO, 2025b). Although non-steroidal anti-inflammatory drugs (NSAIDs) may be considered for pain relief, dengue and other haemorrhagic arboviral infections should first be excluded because of bleeding risk (WHO, 2024b).

However, patients with severe disease, including meningitis, encephalitis, meningoencephalitis, Guillain–Barré syndrome, haemorrhagic manifestations, or pregnancy-associated complications, may require hospital-based supportive care. In such cases, management may include fluid therapy, neurological monitoring, intensive care support, and multidisciplinary follow-up when indicated (Bai et al., 2025; WHO, 2025b). Therefore, early recognition, laboratory confirmation, clinician awareness, and integration of OROV into arboviral surveillance systems are essential to reduce misdiagnosis and strengthen preparedness in regions at risk of future emergence (WHO, 2026).

## Potential Emergence and Risk Factors of OROV in Asia

Taken together, the epidemiological expansion, transmission ecology, clinical overlap with other arboviral infections, and diagnostic limitations provide the basis for evaluating whether OROV could emerge beyond its established range. The following sections discuss these factors in detail to assess the potential emergence and risk factors of OROV in Asia. Although OROV is not currently endemic in Asia, and no confirmed local transmission has been reported, this should not lead to complacency (CDC, 2024). Recent outbreaks in the Americas indicate that OROV is undergoing a major epidemiological shift, leading to the emergence of reassortant lineages. This subsequently led to the OROV expansion and establishment in previously non-endemic areas. These changes suggest that OROV is no longer restricted to its historical ecological niche and should be viewed as a growing arboviral concern for regions outside the Americas (Scachetti et al., 2025).

Future OROV emergence in Asia would likely result from the convergence of several risk factors rather than a single introduction event. Viral adaptability driven by mutation and genome-segment reassortment may allow OROV to adjust to new vector-host systems, while uncertainty regarding vector competence highlights the need to identify the mosquitoes and *Culicoides* species capable of sustaining transmission. At the same time, human mobility, urbanisation, deforestation, climate change, peri-urban expansion, and wildlife-livestock-human interfaces may create ecological opportunities for viral amplification if OROV is introduced (Gallichotte, Ebel & Carlson, 2025).

Asia should therefore not await confirmed local transmission before implementing preventive and preparedness measures. The clinical overlap between OROV and other arboviral infections, particularly dengue, Zika, and chikungunya, may delay its recognition in settings where surveillance of acute febrile illness is already dominated by dengue-like syndromes. This concern is further amplified by recent reports of severe outcomes, including neuroinvasive disease and possible adverse pregnancy outcomes, which suggest that missed transmission could have important public health consequences. This section highlights three urgent and interconnected priorities for Asia: viral adaptability, ecological and zoonotic risk, and human and climate-driven emergence factors (Farias et al., 2025).

### Viral adaptability

**Figure 2:**
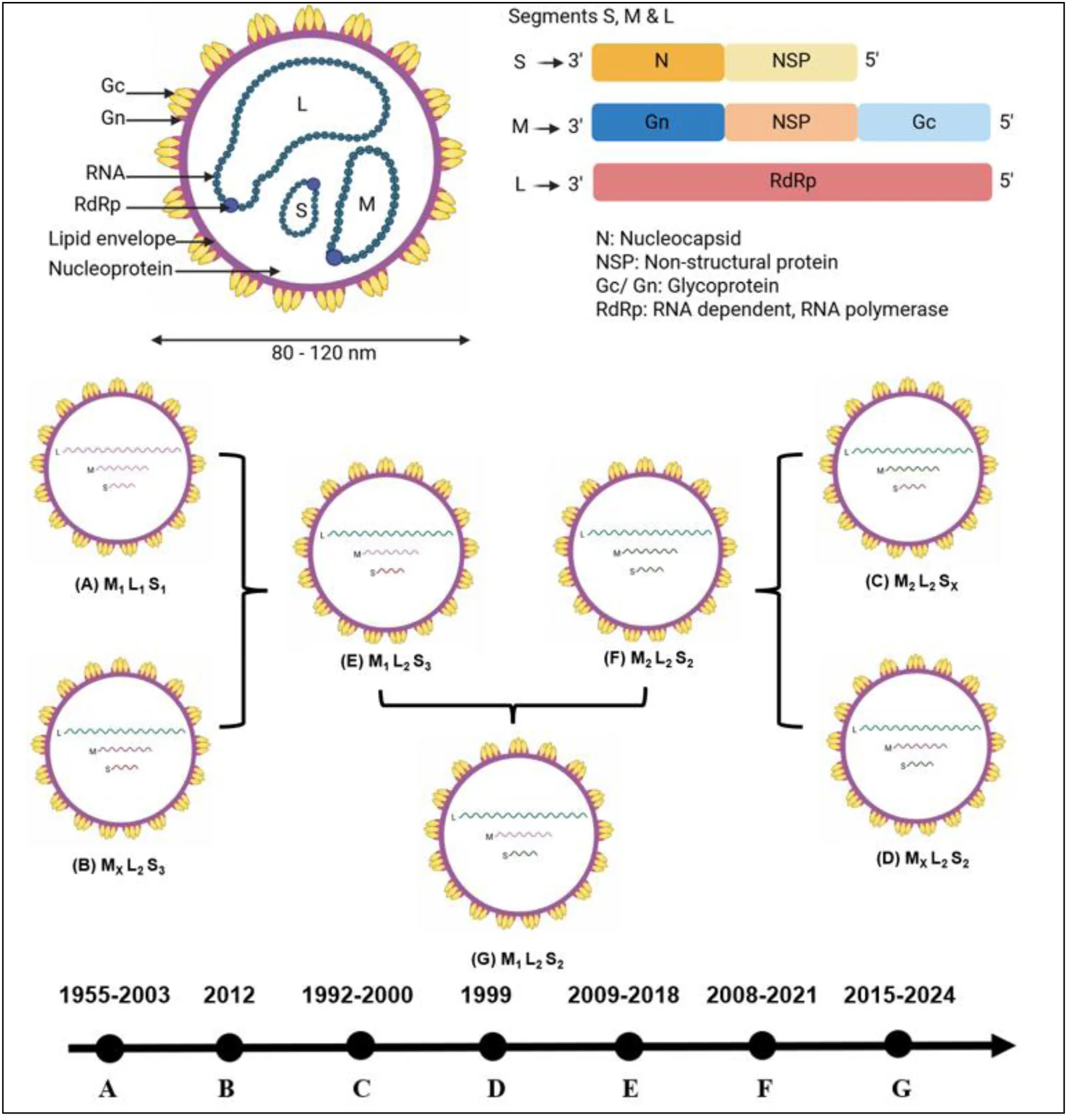
Genome structure and reassortment history of OROV and related *Orthobunyaviruses*. OROV is an enveloped, tripartite negative-sense RNA virus containing small (S), medium (M) and large (L) genome segments that encode the nucleocapsid protein, glycoproteins/non-structural proteins and RNA-dependent RNA polymerase, respectively. Segment reassortment among OROV and related viruses, including PEDV and IQTV, has generated distinct lineages such as BR/TT 1955–2003, PE 1992–2000, BR 2009–2018, PE/CO/EC 2008–2021 and BR 2015–2023, highlighting the evolutionary role of genome reassortment in OROV diversification. Created in: Biorender.com and Microsoft Word.

OROV is a spherical, lipid-enveloped virus with a genome comprising three single-stranded, negative-sense RNA segments: S, M, and L (Briese, Calisher & Higgs, 2013; Elliott, 2014). The S segment encodes the nucleocapsid protein, which packages viral RNA into ribonucleoprotein complexes required for genome stability. The M segment produces a polyprotein that is processed into the envelope glycoproteins Gn and Gc as well as non-structural protein involved in viral assembly and morphogenesis, while the L segment encodes the RNA-dependent RNA polymerase that mediates transcription and replication (Elliott, 2014; Ferron et al., 2017; Gao et al., 2025; Shi et al., 2010). This genome structure underpins OROV adaptability by supporting both mutation accumulation and segment exchange (Fig. 2).

The absence of proofreading activity in the OROV RNA-dependent RNA polymerase increases replication error rates, allowing point mutations to accumulate and contribute to gradual viral diversification (Ding et al., 2025). In addition, the segmented genome permits reassortment when related *Orthobunyaviruses* co-infect the same host or vector cells, generating viruses with different combinations of S, M, and L segments (Briese, Calisher & Higgs, 2013; Elliott, 2014; Gutierrez et al., 2020; Schwartz, 2025; Tilston-Lunel et al., 2017). This process is particularly relevant to the M segment because it encodes Gn and Gc, which are central to host-cell entry and vector interactions. Variation in this segment may therefore influence tissue tropism, antigenicity, vector competence, replication fitness, virulence, and transmission efficiency (Gunter et al., 2026; Gutierrez et al., 2020; Lin et al., 2025; Shi et al., 2010).

Evidence from OROV-related viruses demonstrates that reassortment occurs in natural settings. Iquitos virus carries OROV-derived S and L segments but an M segment from another Simbu serogroup virus. Reassortant origins have also been reported for Madre de Dios virus and Perdões virus, supporting the view that OROV belongs to an actively exchanging *Orthobunyavirus* gene pool rather than a fixed evolutionary lineage (Aguilar et al., 2011; Amorim et al., 2024; Gutierrez et al., 2020; Ladner et al., 2014; Walsh, Robert & Christofferson, 2021).

Recent genomic analyses further suggest that mutation and reassortment have contributed to OROV diversification. Sequences collected from 1955 to 2024 indicate accelerated spatiotemporal evolution, with 2023–2024 outbreak viruses identified as reassortants within distinct Brazil and western Amazon Basin lineages. Structural modelling also suggests that changes in the polymerase and Gc glycoprotein may affect viral fitness and adaptation (Ding et al., 2025). WHO has identified two major reassortant lineages currently circulating in the Americas: BR-2015–2024 in Brazil and Cuba, and PE/CO/EC-2008–2021 in Peru and Ecuador, with both lineages detected in Colombia. The BR-2015–2024 lineage appears to combine an eastern Amazon M segment with L and S segments related to viruses from Peru, Colombia, and Ecuador, and may have emerged in Amazonas before spreading to non-endemic areas, including Cuba (WHO, 2025a).

Outbreak-associated strains may also differ in biological fitness. The AM0088 strain from the 2023–2024 Brazil outbreak has been reported to replicate more efficiently in mammalian cells than earlier strains, suggesting that contemporary OROV lineages may be acquiring traits that improve vertebrate host amplification and epidemic potential (Dias et al., 2024; Scachetti et al., 2025). This interpretation remains preliminary and requires validation in animal and vector models.

Overall, OROV adaptability reflects the combined effects of replication errors and reassortment. For Asia, where diverse vectors, vertebrate hosts, and co-circulating arboviruses are present, this genetic flexibility supports preparedness even though sustained local transmission has not been confirmed. Surveillance should therefore include whole-genome sequencing of all three segments, with emphasis on M-segment divergence, reassortment events, and mutations affecting glycoprotein or polymerase function (Ding et al., 2025; Gutierrez et al., 2020; Tilston-Lunel et al., 2017); WHO, 2025a).

### Ecological and zoonotic risks

The ecological and zoonotic risk of OROV in Asia should not be underestimated simply because the virus has not yet been detected in the region or because its classical Neotropical vector, *Culicoides paraensis*, has not been confirmed. In South and Central America, OROV relies on a flexible, multi-host transmission infrastructure where diverse sylvatic reservoirs and bridge vectors form fluid connections with human populations (Nascimento et al., 2020; Yoosuf et al., 2025). This ecological plasticity strongly suggests that if OROV is introduced into an environment with analogous host-vector networks, it can successfully exploit local interfaces.

Asia possesses several ecological conditions that could support arboviral spillover. The region has high biodiversity, dense human populations, expanding peri-urban landscapes, and frequent wildlife–livestock–human contact. It also supports diverse arboviruses and zoonotic pathogens in underexplored wildlife hosts, including bats, rodents, wild birds, and small mammals. These taxa are important reservoirs, incidental hosts, or amplifying hosts because of their ecological adaptability, mobility, and frequent interaction with human-modified environments (Luis et al., 2013; Olival et al., 2017). Environmental disturbance, habitat fragmentation, and land-use change can increase contact among wildlife, vectors, livestock, and humans, creating conditions that favour spillover from sylvatic cycles into human populations (Plowright et al., 2017; Wang & Anderson, 2019).

Recent wildlife surveillance highlights how easily alternative sylvatic hosts can remain hidden in routine monitoring. For instance, recent studies in Malaysia identified a novel mammalian orthoreovirus and detected dengue virus serotype 2 RNA in the faeces of wild treeshrews (*Tupaia* spp.) (Siew et al., 2026); Siew et al., 2025a). While these findings do not implicate treeshrews as reservoirs for OROV, they serve as a critical proof-of-concept. They provide an example showing that non-classical wildlife hosts may be missed by standard routine surveillance. This is relevant for OROV preparedness because the sylvatic components of OROV ecology remain incompletely defined, and similar surveillance gaps could delay recognition if the virus is introduced into Asia.

The endemic circulation of other Simbu group *Orthobunyaviruses* in Asia, including Akabane and Sathuperi viruses, further supports this concern. Although these viruses differ from OROV in host range, vector specificity, and transmission dynamics, their persistence demonstrates that Asia already has an ecological framework capable of sustaining *Orthobunyavirus* circulation (Kirkland, 2015; Yanase, Murota & Hayama, 2020). This includes suitable vector populations, susceptible vertebrate hosts, permissive environmental conditions, and established vector-host contact networks. Therefore, the key question is not whether Asia exactly replicates the Neotropical OROV transmission system, but whether its existing arboviral infrastructure could provide alternative routes for OROV adaptation, amplification, and spillover.

Furthermore, the absence of *Culicoides paraensis* in Asia does not rule out the possibility of OROV transmission, as the region has a highly diverse biting midge fauna. Other local *Culicoides* species may potentially fulfil a similar vector role if they can support OROV transmission, dissemination, and onward spread. Several species, including *Culicoides oxystoma*, *Culicoides imicola*, and *Culicoides brevitarsis*, are widely distributed across Asia and are recognised vectors of animal arboviruses, particularly livestock-associated viruses such as bluetongue virus and Akabane virus (Carpenter, Wilson & Mellor, 2009; Mellor, Boorman & Baylis, 2000). Their involvement in established arboviral transmission cycles indicates that an ecological framework for the maintenance of midge-borne viruses already exists in the region. These species should therefore be prioritised for experimental evaluation rather than dismissed because the primary Neotropical vector has not been confirmed in Asia.

Alternative mosquito vectors could also potentially support OROV transmission in Asia. *Culex quinquefasciatus* has been proposed as a potential secondary vector of OROV and is widely distributed across Southeast Asia (Gallichotte, Ebel & Carlson, 2025; Walsh, Robert & Christofferson, 2021). Although current evidence suggests that its competence for OROV transmission may be limited, vector competence should not be interpreted as a fixed biological barrier since historical evidence from arbovirology suggests that vector barriers can be reshaped by viral mutation, reassortment, ecological pressure, and repeated vector exposure (Tsetsarkin et al., 2007; Vazeille et al., 2007). As such, future changes could alter viral replication or dissemination efficiency in *Culex quinquefasciatus*, potentially transmitting the virus from imported cases to the local population. In Asia, where *Culex* mosquitoes and diverse *Culicoides* species occur across urban, peri-urban, agricultural, and livestock-associated environments, OROV would encounter a broad vector landscape in which adaptation or alternative vector recruitment cannot be excluded (Yanase, Murota & Hayama, 2020). This concern should be addressed before OROV gains ecological and molecular compatibility with Asian transmission systems.

Therefore, Asia should be considered an ecologically receptive region for potential OROV introduction and amplification. Ongoing rapid urbanisation, deforestation, agricultural expansion, climate variability, and habitat fragmentation are reshaping vector distribution and host-vector interactions across the region (Jones et al., 2008; Plowright et al., 2017). Collectively, these pressures create One Health risk zones where wildlife reservoirs, domestic animals, biting arthropods, and human populations increasingly overlap. Because human and wildlife populations in Asia are likely to have limited pre-existing immunity to OROV, viral introduction into densely connected sylvatic–urban interfaces could increase the risk of sustained spillover rather than isolated transmission events.

Consequently, Asia’s ecological and zoonotic landscape should be regarded as potentially vulnerable to OROV emergence and amplification. Successful establishment would still depend on complex interactions among viral adaptation, vector competence, host availability, and environmental suitability (Santos et al., 2025; WHO, 2024b; WHO, 2025a). Nevertheless, these uncertainties underscore the need for strengthened preparedness rather than diminishing the perceived risk. Targeted entomological, ecological, virological, wildlife, livestock, and seroepidemiological surveillance is therefore urgently needed to determine whether OROV could exploit Asia’s existing transmission infrastructure before silent spillover progresses into sustained local transmission.

### Human and climate drivers

Human activities and environmental changes could accelerate viral introduction, adaptation, and establishment in new regions. Land-use change, deforestation, rapid urbanisation, agricultural expansion, and global mobility have historically driven the geographic expansion of arboviral infections such as dengue, chikungunya, and Japanese encephalitis across Asia (Da Rosa et al., 2017; Gomes, Rebelo & Alves de Sousa, 2022; Sakkas et al., 2018; Scachetti et al., 2025). These cases suggest that arboviral emergence is rarely accidental; it occurs when viral evolution intersects with human-modified environments, dense populations, and efficient vector networks. *Aedes aegypti*, for example, was introduced from Africa and became established across Asia, fuelling major dengue outbreaks, while chikungunya resurgence was intensified by viral mutations that enhanced transmission through *Aedes albopictus* (Bhattacharya, Chatterjee & Tilak, 2025; Näslund et al., 2021).

For OROV, this process of global geographic expansion has already begun. Imported OROV cases reported in the United States and Europe, including cases linked to travel from Cuba and Brazil, demonstrate that the virus could move through international transit networks *via* infected travellers (Dias et al., 2024; Morrison et al., 2024). Unlike many temperate settings, Asia contains highly populated tropical and subtropical urban centres, dense peri-urban settlements, abundant biting vectors, and extensive human-animal-environment interfaces. As one of the world’s major hubs for international travel, trade, labour movement, and tourism, Asia provides multiple entry points through which viraemic travellers could introduce OROV into environments suitable for local amplification. Thus, travel-associated cases should not be viewed as isolated epidemiological events, but as evidence that intercontinental viral movement is possible.

Climate change may further lower the ecological barriers to OROV establishment by increasing the suitability of vector-borne transmission in parts of Asia. Rising temperatures, rainfall variability, humidity, monsoon-driven water accumulation, and shifting seasonal patterns can increase vector abundance, extend vector survival, expand geographic ranges, and increase biting frequency (Da Rosa et al., 2017; De Mendonça et al., 2021; Guagliardo et al., 2024; Burkett-Cadena, 2025; Sakkas et al., 2018; Scachetti et al., 2025; Walsh, 2021). Warmer and more humid conditions may also shorten the extrinsic incubation period, allowing vectors to become infectious more rapidly and increasing the probability of transmission during their lifespan. Therefore, climate change not only expands vector distribution; it can also increase the efficiency of virus-vector interactions and lower the biological threshold for local transmission (Abbasi, 2025; Terradas et al., 2024; Zain et al., 2024).

This risk is particularly relevant in South and Southeast Asia, where monsoon rainfall, persistent humidity, rapid land-use change, and agricultural water systems already generate favourable vector habitats. Under warming conditions, *Aedes aegypti* may show greater thermal resilience and reproductive potential, while *Culicoides* midges can thrive in humid, rain-fed, organic-rich environments common in monsoon-influenced regions (Abbasi, 2025; Elbers, Koenraad & Meiswinkel, 2015). Monsoon variability in India has been associated with stagnant water accumulation and increased vector proliferation, illustrating how climate instability can intensify vector exposure in densely populated regions (Bowden, Foster & Parkes, 2023). In this context, rice cultivation, irrigation canals, livestock watering areas, and household or farm water storage are best interpreted as ecological amplifiers of climate effects. These systems can retain water after heavy rainfall or during drought, extending larval habitat availability and sustaining vector populations near human settlements (Dias et al., 2024; Rocklöv & Dubrow, 2020; Tyagi et al., 2023).

Environmental changes across Asia create important parallels with the Amazon Basin, where OROV is endemic, as both regions comprise warm, humid, rainfall-driven ecosystems that can support vector-borne virus transmission (Subissi et al., 2026). Although the risk may be highest in tropical and subtropical areas such as Southeast Asia, South Asia, and southern China, climate change may expand ecological suitability into other parts of Asia by altering rainfall patterns, increasing flood and drought cycles, and extending vector activity. The International Panel on Climate Change (IPCC) reports that rising temperature and heavy rainfall are expected to increase the risk of vector-borne diseases, including dengue and malaria, in tropical and subtropical Asia, supporting the broader relevance of climate-sensitive arboviral risk beyond Southeast Asia alone (IPCC, 2022). Climate change has also been linked to shifts in the transmission season and geographic distribution of infectious diseases in Asia, particularly through rising temperature, irregular rainfall, and changes in vector ecology (Zain et al., 2024). Therefore, OROV risk should be framed as a wider regional preparedness issue shaped by climate variability, land-use change, vector ecology, and human mobility, rather than as a concern limited to Southeast Asia.

Taken together, human mobility and climate change render the potential emergence of OROV in Asia a foreseeable public health preparedness challenge rather than a remote theoretical risk. Travel-associated infections reported in the United States, Canada, and Europe demonstrate that OROV can move beyond endemic regions through international travel, while recent outbreaks have also involved areas where the virus had not previously been reported, including Cuba and non-endemic areas of South America (Morrison et al., 2024; PAHO, 2025a; WHO, 2024a). At the same time, rising temperature, heavy rainfall, humidity, monsoon-driven water accumulation, agricultural irrigation, livestock activity, urbanisation, deforestation, and land-use change may reshape vector-host-human interfaces across Asia by increasing vector abundance, prolonging vector activity, and intensifying human exposure (Zain et al., 2024). When these conditions converge with international travel, dense populations, abundant vectors, delayed diagnosis, and established arboviral systems, an imported OROV case could progress from isolated introduction to local transmission before being clinically recognised. The critical question is therefore not whether OROV can reach Asia, but whether regional surveillance, diagnostic capacity, and vector monitoring can detect and contain it before silent establishment occurs.

## Conclusions

OROV is increasingly recognised as an emerging arboviral pathogen with expanding epidemiological, clinical, and evolutionary significance. Recent geographic expansion beyond traditional endemic areas, the emergence of reassortant lineages, travel-associated cases, and reports of neurological disease, adult fatalities, and vertical transmission indicate that OROV is no longer a geographically confined Amazonian infection. Its segmented RNA genome, reassortment capacity, and evidence of contemporary lineage diversification provide a virological basis for adaptability, while unresolved gaps remain in vector competence, reservoir ecology, transmission efficiency, and determinants of severe disease.

For Asia, the major concern is not a single isolated risk factor, but the synergistic convergence of viral adaptability, suitable vector-host ecology, climate-sensitive environments, dense human populations, and global mobility. Although no confirmed autochthonous OROV transmission has been reported in Asia, this absence should be interpreted cautiously because dengue-like clinical overlap, limited molecular testing, and insufficient entomological surveillance may delay recognition. If introduced into permissive ecological settings, OROV could encounter diverse *Culicoides* species, widespread *Culex* mosquitoes, wildlife–livestock–human interfaces, and climatic conditions that may support amplification.

Preparedness should therefore move beyond passive observation, for example, by integrating One Health surveillance. These activities include incorporating OROV into arboviral diagnostic panels, strengthening surveillance in humans and travel-linked case detection, expanding genomic surveillance of all three viral segments, and conducting reservoir-focused surveillance in wildlife, livestock, and peri-domestic animals. Additionally, vector competence studies involving regionally relevant biting midges and mosquitoes are essential to strengthen preparedness efforts. Collectively, these activities could help to determine whether Asian ecosystems could support OROV transmission and help prevent future outbreaks.

## Additional Information and Declarations

### Data Availability

The following information was supplied regarding data availability: This is a Literature Review.
